# Chronic Ogilvie Syndrome Revealed During Postoperative Rehabilitation

**DOI:** 10.7759/cureus.99067

**Published:** 2025-12-12

**Authors:** Brittany E Reid, Mario Perez, Katharine Balbuena

**Affiliations:** 1 Rehabilitation Medicine, Independent Researcher, Chicago, USA; 2 Osteopathic Medicine, Edward Via College of Osteopathic Medicine, Monroe, USA; 3 Internal Medicine, Independent Researcher, Mobile, USA; 4 Physical Medicine and Rehabilitation, Willis Knighton Rehabilitation, Shreveport, USA

**Keywords:** acute colonic pseudo-obstruction, autonomic nervous system dysfunction, chronic intestinal pseudo obstruction (cipo), gastrointestinal motility disorders, inpatient rehabilitation, ogilvie's syndrome, physical medicine and rehabilitation

## Abstract

Ogilvie's syndrome, or acute colonic pseudo-obstruction, is a rare condition characterized by colonic dilation without mechanical obstruction. While often seen acutely in hospitalized or postoperative patients, chronic forms are also possible. We present a case of a 73-year-old male who was admitted to inpatient rehabilitation following a posterolateral fusion at C2-T1 with decompressive cervical laminectomy, facetectomy, and foraminotomies. His medical history included cervical spondylosis with radiculopathy, gastroesophageal reflux disease (GERD), chronic constipation, and prior lumbar and cervical spine surgeries. Upon admission, he reported persistent nausea, abdominal distension, constipation, and neck pain. Physical examination revealed a nontender, firm, and distended abdomen with high-pitched bowel sounds. A kidney, ureter, bladder X-ray demonstrated colonic dilation consistent with Ogilvie's syndrome. A review of past imaging from 11 years prior confirmed chronic and previously unmanaged pseudo-obstruction. Conservative management with laxatives led to gradual improvement without surgical or pharmacologic intervention. Functional recovery was achieved with rehabilitation, and his discharge plan included follow-up with a gastrointestinal physician and home health services. This case highlights the importance of recognizing potential contributors to postoperative gastrointestinal symptoms. Early imaging review, conservative bowel management, and specialist follow-up are key in these complex patients.

## Introduction

Acute colonic pseudo-obstruction (ACPO), or Ogilvie’s syndrome, is a rare condition defined as colonic obstruction without a mechanical cause [1-5]. Its incidence is estimated at 100 per 100,000 patients, with an overall mortality of 8-9.4% that rises to nearly 50% when complicated by perforation requiring surgery [1,2,4,6]. Prompt recognition and treatment, therefore, improve survival [1,2,4-6].

The pathophysiology is thought to involve autonomic imbalance, with reduced parasympathetic and possibly increased sympathetic activity in the colon [1]. Clinically, ACPO presents with increased bowel sounds, right-sided abdominal distention, nausea, vomiting, constipation, and sometimes, electrolyte abnormalities such as hypokalemia [3,4,6-8]. It may mimic postoperative ileus; however, ileus usually develops within one to three days postoperatively, whereas ACPO often presents after three to five days and is characterized on imaging by isolated large-bowel dilation [5,8]. Risk factors include advanced age, chronic illness, and recent surgery, particularly orthopedic, gynecologic, transplant, or urologic procedures [1,3,4,9]. Providers should also order a colonoscopy/water-soluble contrast enema or non-contrast computed tomography (CT) of the abdomen to rule out any mechanical cause [9]. Management depends on the degree of colonic dilation; patients with diameters under 9 cm may be managed conservatively with fluids, electrolyte correction, and bowel rest, while neostigmine is effective for refractory cases [1,7,8,10]. If ineffective, decompression with a rectal tube or endoscopy may be attempted. Severe dilation (≥9 cm) may require surgical decompression or cecostomy [7-8].

In rare cases, ACPO may evolve into chronic intestinal pseudo-obstruction (CIPO), defined as persistent or recurrent pseudo-obstruction without a mechanical cause for at least six months [11]. CIPO is most often diagnosed in infancy or young adulthood. However, adult-onset cases also occur, often secondary to neuromuscular or autonomic dysfunction [11-14]. Patients present with recurrent abdominal pain, distention, nausea, vomiting, and constipation [11-14]. Diagnosis is challenging and often delayed for years [12,14]. While there is no recognized algorithm, it is recommended to order an abdominal X-ray and manometry studies [11,14,15]. The abdominal X-ray will show similar findings to a small bowel obstruction: dilated bowel loops with or without air fluid levels and potentially a dilated stomach [11,15]. CT abdomen with contrast should also be performed to exclude mechanical causes of obstruction [11,14]. There is debate on the utility of manometry studies [14], but these studies can assist in the diagnosis of CIPO in certain cases by assessing the strength and coordination of the gastrointestinal (GI) system [11]. Biopsies of the intestine can be used to classify the type of CIPO [11,12]. Given this, clinical experience is still the primary way to identify these cases [14].

Treatment is primarily supportive, focusing on nutritional optimization, symptom control, and avoidance of opioids. Prokinetic medications, neostigmine, and pyridostigmine may provide benefits such as increased GI motility [11-13,16]. For pain, it is recommended to avoid opiates and instead prescribe gabapentin [11,13] and tri-cyclic antidepressants [11]. In refractory cases, surgical interventions or intestinal transplantation may be considered [13,16], although both carry risks of GI adhesions, which can worsen dysmotility [11,16]. Intestinal transplant provides up to 80% of symptomatic improvement, with a graft survival rate between 45-83% [16]. Another study showed that the three-year survival rate after a small bowel transplant was 66% [14]. Nutritional support is critical; approximately 60-80% of patients require parenteral nutrition [11,13,14]. Adequate nutrition can be difficult to achieve for these patients due to their tendency to experience GI obstructions, and up to 30% of patients suffer from small intestinal bacteria overgrowth (SIBO) [11,14,16]. It is recommended to monitor for vitamin B12 and fat-soluble vitamin deficiencies in patients with SIBO due to the effect on small intestine absorption.

Here, we present a patient initially diagnosed with ACPO but with symptoms and imaging findings dating back 11 years, raising concern for an underlying diagnosis of CIPO. This case highlights the critical role of rehabilitation clinicians in recognizing atypical GI dysfunction that may be overlooked in acute care, where recognition and coordinated management can prevent complications and improve recovery.

## Case presentation

A 73-year-old male presented to inpatient rehabilitation after receiving a posterolateral fusion at C2-T1 with decompressive cervical laminectomy, facetectomy, and foraminotomies in the spring of 2025. The patient was experiencing numbness, tingling, weakness, and left upper extremity pain prior to the surgery. His past medical history included gastroesophageal reflux disease (GERD), hearing loss, chronic constipation, hemorrhoids, hiatal hernia, stomach ulcer, cervical spondylosis with radiculopathy, and cervical stenosis of the spinal canal. Past surgical history included a fusion of L5-S1, two anterior cervical spinal fusions, and colon surgery. Family history was unremarkable. 

His social history was significant for being a former 10-pack-per-year smoker for 16 years; his quit date was over 15 years ago at the time of admission. Alcohol use included hard liquor, and frequency of use was classified as monthly or less. He denied any illicit drug use. The patient had no known drug allergies. His current medications included gabapentin 300 mg every four hours as needed for neuropathic pain, hydrocodone-acetaminophen 10 mg-325 mg one per day as needed for pain, and esomeprazole magnesium 40 mg once a day as needed for acid reflux. For rehabilitation purposes, the patient lived alone in a one-story house. 

**Figure 1:**
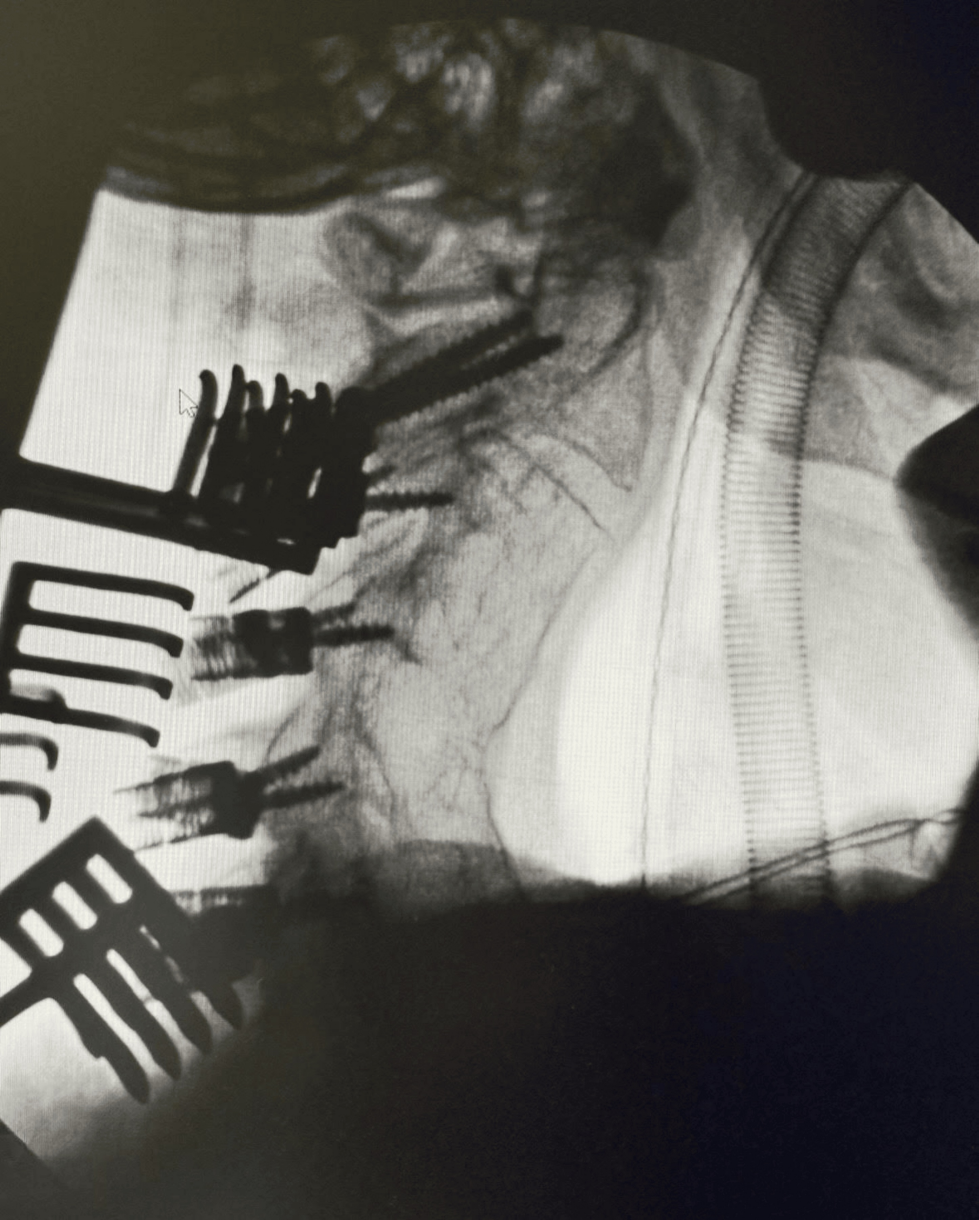
Cervical spine fluoroscopy taken before rehabilitation showed extensive fusion hardware and postoperative changes. Multiple screws are visible, illustrating the complexity of the patient’s surgical history.

The review of systems was positive for nausea, constipation, neck pain, and left arm numbness. Vital signs were normal with a temperature of 98.3℉, a pulse of 75 beats per minute, respirations 16 breaths per minute, blood pressure 118/82 mmHg, and oxygen saturation of 98% on room air. Physical examination revealed a posterior surgical incision on the cervical spine with a drain site with some drainage. The incision appeared to be healing as expected, with no evidence of infection. The patient experienced some cervical spine tenderness, pain, and decreased range of motion. The exam was also notable for essential tremor and some ulnar numbness in the left hand. Strength was 4/5 in the upper extremities and 5/5 in the lower extremities. GI examination revealed a non-tender, distended, and firm abdomen with hypoactive bowel sounds. The rest of the exam was normal. Laboratory results from hospital discharge showed the findings in Table 1, including postoperative anemia. Other laboratory studies, including lactate, were within normal limits. Urine analysis on admission was unremarkable. No new imaging was done prior to rehabilitation admission, other than a cervical spine X-ray and fluoroscopy, which showed findings consistent with postoperative changes (Figure 1).

**Table 1:** Abnormal laboratory findings upon admission to the rehabilitation hospital, showing postoperative anemia.

Test	Value	Reference Range
Hemoglobin	11.7 g/dL	13.1-16.8 g/dL
Sodium	135 mmol/L	137-145 mmol/L
Glucose	113 mg/dL	70-109 mg/dL

Upon admission to the rehabilitation hospital, he experienced continued nausea, constipation, pain, and stiffness in the neck with functional decline. He denied any abdominal pain or bowel movements. He was started on senna-s for his constipation, and methocarbamol was added for neck pain and stiffness. The next day, he had continued constipation, and his abdomen was still nontender, distended, and firm with poor bowel sounds. Due to his symptoms and physical examination, a kidney, ureter, bladder X-ray (KUB) was ordered, and the dose of senna-s was doubled.

**Figure 2:**
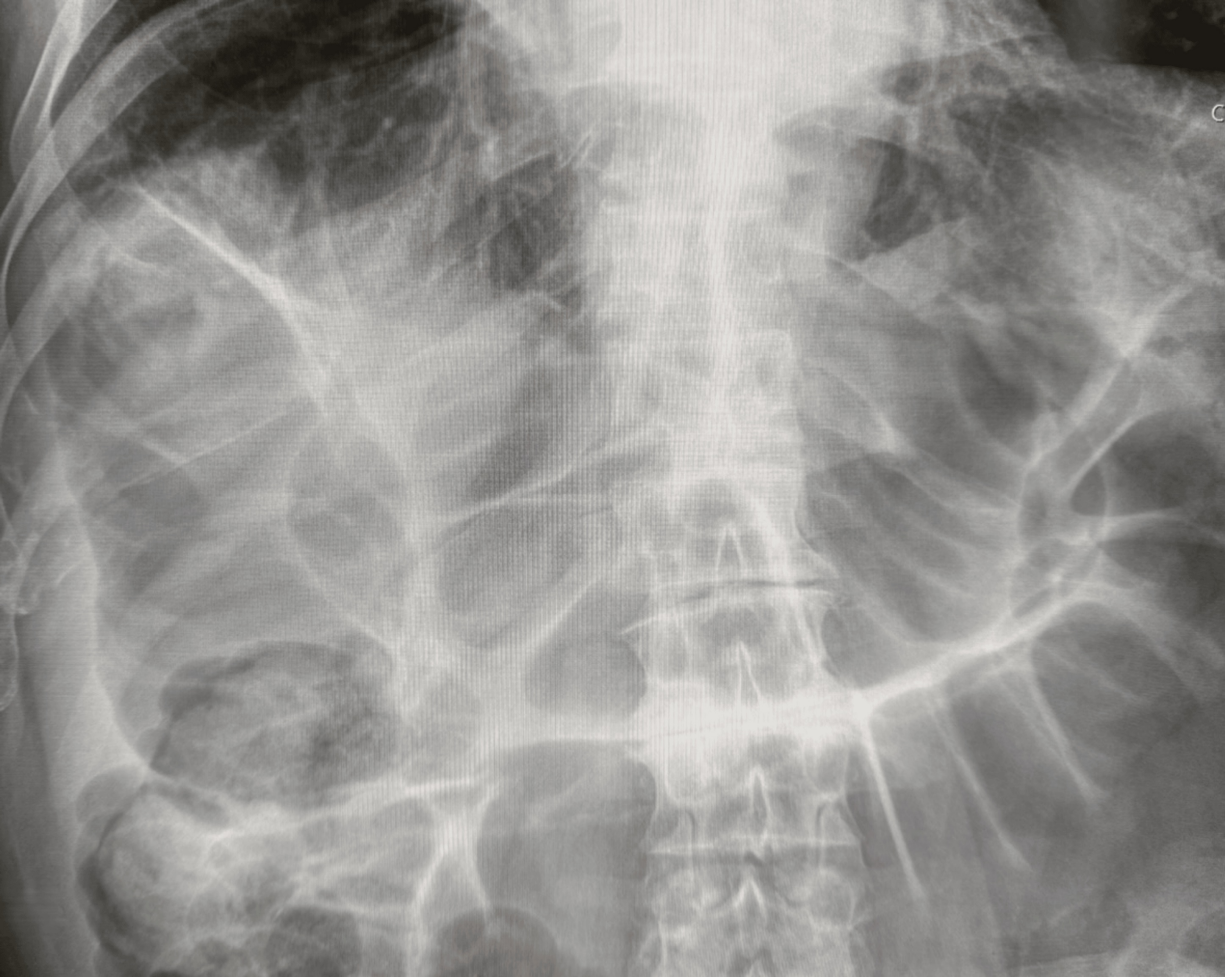
Kidney, ureter, bladder (KUB) radiograph at the rehabilitation hospital showed diffuse dilation of the large intestine without a clear transition point. The marked colonic distension is an important clue for Ogilvie's syndrome.

**Figure 3:**
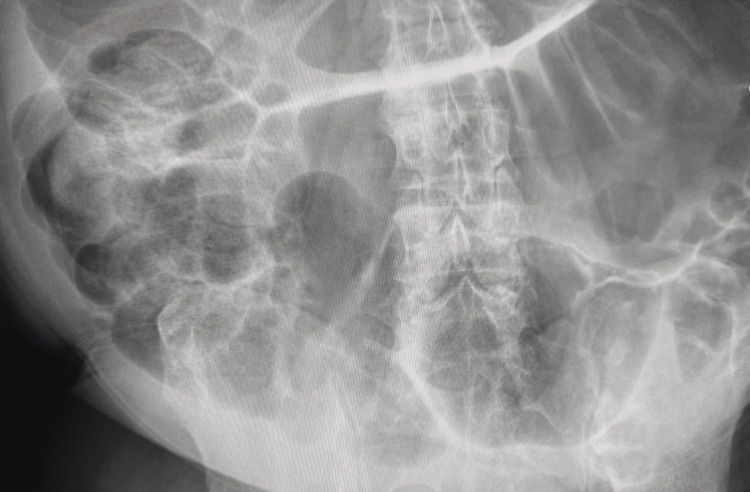
Additional kidney, ureter, bladder (KUB) radiograph in rehabilitation, taken from a different angle, also demonstrated significant colonic dilation without an obstructive transition point. The repeated finding across views reinforces the diagnosis of pseudo-obstruction rather than a focal blockage.

**Figure 4:**
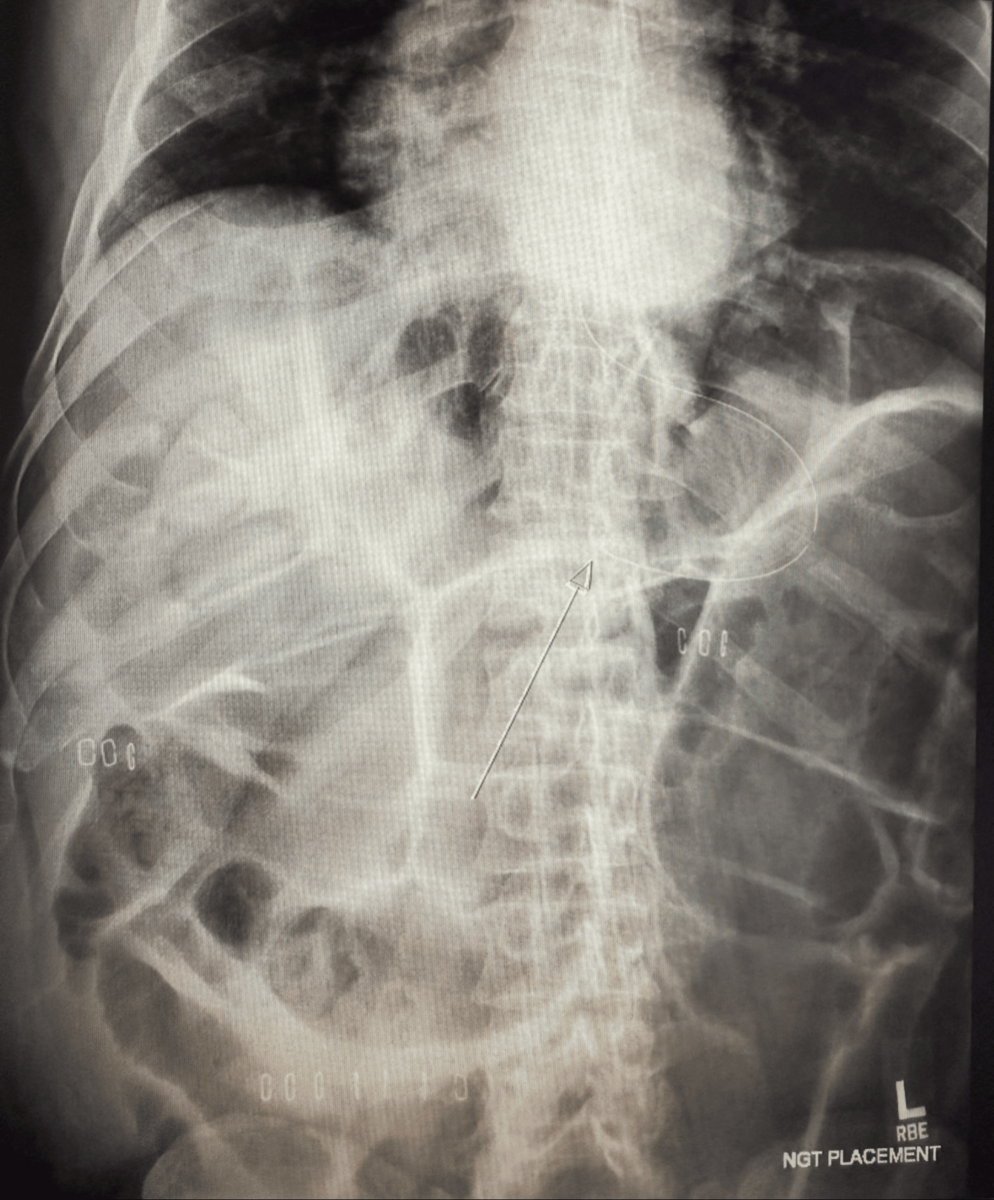
Kidney, ureter, bladder (KUB) from 11 years prior to admission showed features of Ogilvie’s syndrome, including marked bowel dilation with a nasogastric tube in place (arrow). This historical comparison highlights the chronic nature of the patient’s condition.

The next day, the patient still had some pain in the neck, but no nausea, vomiting, or abdominal pain. He reported some bowel movements since the previous day. His examination revealed a nontender, distended abdomen with high-pitched bowel sounds in the left upper and lower quadrants. The abdomen was softer than the previous day, but still firm. The KUB revealed findings consistent with Ogilvie's syndrome (Figures 2-3). When viewing the updated imaging, a previous X-ray from 11 years prior also revealed similar findings, and he was diagnosed with Ogilvie's syndrome (Figure 4). The patient denied following this diagnosis with a specialist or receiving any interventions to resolve his symptoms. The persistent findings on X-ray from 11 years ago to this admission indicated chronic Ogilvie's syndrome. He was then started on daily lactulose. Due to the imaging confirming chronic Ogilvie's syndrome, the improvement of symptoms and examination with the laxative, monitoring was continued in rehabilitation.

Two days later, the patient continued to have improvement in his abdominal distension. However, the patient still denied having a good bowel movement. Due to this, magnesium citrate was added to his bowel regimen. The next day, the abdominal distension continued to improve. He continued to do well in therapy and was a minimal-moderate assist for standing balance with a right lean noted. The next day, he continued to improve in function; however, he still denied having a good bowel movement. A GI follow-up was discussed for discharge, and the patient concurred since he was previously unaware of his diagnosis requiring a specialist. Concerns for discharge were addressed regarding living alone and needing assistance for obtaining and preparing food, due to his postoperative driving limitations. Eleven days into admission, laboratory results were repeated and demonstrated improved glucose and anemia (Table 2). All other laboratory values were unremarkable. He had ongoing improvement and reached transfer goals for discharge. Goals were set for his outpatient rehabilitation and follow-up appointments with neurosurgery, GI, and his primary care physicians were scheduled.

**Table 2:** Laboratory findings on day 11 of rehabilitation, showing improved postoperative anemia.

Test	Value	Reference Range
Hemoglobin	12.2 g/dL	13.1-16.8 g/dL
Sodium	136 mmol/L	137-145 mmol/L
Glucose	97 mg/dL	70-109 mg/dL

After 13 days in inpatient rehabilitation, he was discharged home with home health. His discharge medications included methocarbamol 500 mg three times per day for neck stiffness, acetaminophen 325-650 mg every four hours as needed for pain, oxycodone-acetaminophen 10-325 mg every six hours as needed for pain, gabapentin 300 mg every four hours as needed for neuropathic pain, polyethylene glycol 17 g once per day as needed for constipation, lactulose 10 g/15 mL solution 20 g once per day for constipation, esomeprazole magnesium 40 mg once a day as needed for acid reflux, and metoprolol succinate 25 mg at bedtime. On follow-up, the patient attended his outpatient appointments with GI, primary care, and his spine surgery team. He participated in outpatient physical therapy, demonstrating continued improvement in mobility and functional independence. During this period, he developed a postoperative wound complication, which was appropriately managed in the hospital. He was then subsequently discharged in stable condition and continued to do well.

## Discussion

Our patient has a history of ACPO that appears to have transitioned to CIPO. In this discussion, we review his management during hospitalization, compare it to recommended approaches in the literature, and highlight long-term considerations for prognosis and prevention of recurrence.

In terms of inpatient management, the patient was receiving opioids prior to admission, which is generally discouraged in patients with CIPO due to their inhibitory effects on gut motility [11,13]. He continued to require opioids during admission and after discharge for postoperative pain control, making constipation a predictable complication. Clinicians must weigh the benefits of pain control against the risks of worsened dysmotility and should clearly counsel patients on these trade-offs. Alternative options such as gabapentin [11,13] or low-dose tricyclic antidepressants [11,14] are recommended for chronic pain management in CIPO, but given the severity of this patient’s postoperative pain, these agents did not provide sufficient relief. In such cases, aggressive bowel regimens and close monitoring for worsening constipation become essential.

Prokinetic medications would also be reasonable to consider, as the patient experienced recurrent constipation and impaired gut motility. Evidence supports the use of agents such as neostigmine or pyridostigmine [11-13], with emerging data for prucalopride, a 5-HT4 receptor agonist [14]. Macrolides like erythromycin have also been used; however, their role in this patient may have been limited by potential interactions with opioids. Macrolides inhibit the CYP3A4 enzyme, which is the primary metabolic pathway for many opioids, including the oxycodone used to treat the patient [17]. Thus, using macrolides and opioids together raises concerns for an increase in opioid levels, which could exacerbate adverse effects such as reduced motility, sedation, and respiratory depression. Furthermore, a systematic review found that prokinetic agents have not consistently demonstrated benefit in the long-term management of CIPO [14]. Given this combination of limited efficacy data and the patient’s concurrent opioid use, the decision to withhold macrolide prokinetics in this case appears supported.

From a nutritional standpoint, the patient’s ability to resume oral intake is favorable, as this is strongly preferred over parenteral feeding. Despite this, up to two-thirds of patients with CIPO eventually require some form of enteral or parenteral support [11,13,14]. Although parenteral nutrition may be required in certain cases, its high risk of complications highlights the importance of effective dietary management [11]. Current recommendations include small, frequent meals that are low in fat and fiber to minimize symptoms [13,14]. Referral to a nutritionist should be considered early, as patients with CIPO often struggle with unpredictable symptom flares, malabsorption, and recurrent nutritional deficiencies [14].

Long-term management also requires careful planning to reduce recurrence risk, including avoiding medications that impair motility (particularly opioids and anticholinergics) [11,13,14] and evaluating for small intestinal bacterial overgrowth [11,14]. Maintaining regular follow-up with gastroenterology and nutrition specialists is also recommended [12,14,15]. Intestinal transplantation remains a last resort, reserved for patients with severe, refractory disease who fail medical and nutritional therapy [13,15].

Overall, the management in this case aligns with published literature but underscores the importance of individualized treatment decisions in rehabilitation, especially when comorbid conditions necessitate opioid use. Long-term follow-up with a multidisciplinary team, including gastroenterology, pain management, and a dietitian, is critical to optimize quality of life and reduce recurrence.

## Conclusions

The integration of symptom monitoring, physical examination, and imaging led to the recognition of undiagnosed chronic Ogilvie's syndrome during this patient’s postoperative rehabilitation stay. This underscores the unique opportunity rehabilitation clinicians have to identify medical conditions that may be missed in acute care. Emphasizing accurate diagnosis, individualized management, and careful attention to bowel regimens can meaningfully influence the course of rehabilitation. Ongoing coordination with specialists after discharge is also critical to sustaining recovery and preventing recurrence.

## References

[1] Wells CI, O'Grady G, Bissett IP (2017). Acute colonic pseudo-obstruction: a systematic review of aetiology and mechanisms. World J Gastroenterol.

[2] Tellambura M, Cumberbatch M, Goad J (2022). A case of acute-colonic pseudo-obstruction (Ogilvie syndrome) post robot-assisted radical prostatectomy. Urol Case Rep.

[3] Aguiar D, Fracasso T, Lardi C (2022). Fatal Ogilvie's syndrome after hip surgery and review of the literature. Forensic Sci Med Pathol.

[4] Sunda U, Makwana R, Shaily V, Bhosle S, Choudhary S (2024). Acute colonic pseudo-obstruction or Ogilvie's syndrome - a rare complication in the postnatal period: a case report. J Family Med Prim Care.

[5] Buchanan L, Tuma F (2025). Postoperative ileus. StatPearls.

[6] Mohammad M, Alsheikh K, Madlaji SE, Brimo Alsaman MZ (2025). An Ogilvie's syndrome: a rare case of large bowel pseudo-obstruction. Int J Emerg Med.

[7] Alnasarat A, Elrazzaz M, Manasrah N (2024). A challenging case of recurrent Ogilvie syndrome: exploring causes and treatment modalities. Case Rep Gastrointest Med.

[8] Du C, Iftikhar N, Ganti L, Smith-Gonzalez A (2024). Acute colonic pseudo-obstruction: a case of Ogilvie syndrome. Cureus.

[9] Singh S, Kalshetty K, Pelluru SS, Dey S (2022). Ogilvie's syndrome as a post-operative complication following craniotomy: a case report and literature review. Indian J Anaesth.

[10] Pirouz MS, Tayebi A, Sheibani F, Olamaeian F (2025). Post cesarean section peritonitis: a case report of Ogilvie's syndrome. Clin Case Rep.

[11] El-Chammas K, Sood MR (2018). Chronic intestinal pseudo-obstruction. Clin Colon Rectal Surg.

[12] Radocchia G, Neroni B, Marazzato M (2021). Chronic intestinal pseudo-obstruction: is there a connection with gut microbiota?. Microorganisms.

[13] Piao X, Ying GW, Chaney MJ, Samuel S, Sharko A, Zahra F (2021). Chronic idiopathic intestinal pseudo-obstruction. Cureus.

[14] Zhu CZ, Zhao HW, Lin HW, Wang F, Li YX (2020). Latest developments in chronic intestinal pseudo-obstruction. World J Clin Cases.

[15] Dalby C, Shen T, Thélin C, Ganam S, Velanovich V, Sujka J (2025). Surgical and therapeutic interventions for chronic intestinal pseudo-obstruction: a scoping review. J Neurogastroenterol Motil.

[16] Trimarchi R, Visalli C, Quartararo C, Lucanto MC, Nardo GD, Turiaco N, Salamone I (2021). Radiological evaluation of a case of chronic intestinal pseudo-obstruction (CIPO). Radiol Case Rep.

[17] Liukas A, Hagelberg NM, Kuusniemi K, Neuvonen PJ, Olkkola KT (2011). Inhibition of cytochrome P450 3A by clarithromycin uniformly affects the pharmacokinetics and pharmacodynamics of oxycodone in young and elderly volunteers. J Clin Psychopharmacol.

